# Kuakini HHP Center for Translational Research on Aging: Aims and Preliminary Findings

**DOI:** 10.1093/geroni/igab046.1415

**Published:** 2021-12-17

**Authors:** Bradley Willcox, Richard Allsopp, Peter Martin

## Abstract

Kuakini Medical Center (Kuakini) is creating an interdisciplinary Hawai’i-based Center for translational research on aging. This Center will build upon Kuakini’s five-decades of NIH-funded research, its 420,000-specimen biorepository, and existing strengths in aging research, notably, the 56-year ongoing Kuakini Honolulu Heart Program cohort study (Kuakini HHP), Kuakini Honolulu-Asia Aging Study (Kuakini HAAS), and Kuakini HHP Offspring Study. The overall goal is to find practical means to enhance healthy human lifespan (healthspan). Four research project leaders (RPLs) have been selected from various disciplines for mentorship in translational aging research. The first RPL presentation will introduce a novel mouse model, enabling controlled expression of the pro-longevity gene FoxO3, and assess the impact on lifespan and healthspan phenotypes in mice. These phenotypes will be compared to similar phenotypes in humans with/without the FOXO3 longevity genotype. The second RPL presentation will assess the relation between leukocyte telomere attrition rates (from banked blood collected at three time points over 20-plus years) in older Kuakini HHP men with/without the FOXO3 longevity genotype. The third RPL presentation will assess whether FOXO3 genotype, peripheral leukocyte telomere dynamics (attrition rate, telomerase activity) and inflammatory cytokines mediate the human brain integrity and function with age. This project will utilize structural and functional MRI data from male and female Kuakini HHP Offspring Study participants. The fourth RPL presentation will assess whether APOE e2, e4, and FOXO3 longevity-associated alleles impact 34-year incidence of intracerebral hemorrhage. We will summarize the findings, address the healthspan implications and provide future directions. Supported by NIH 5P20GM125526.

